# Hypercalcemia in Upper Urinary Tract Urothelial Carcinoma: A Case Report and Literature Review

**DOI:** 10.1155/2013/470890

**Published:** 2013-02-13

**Authors:** Keiko Asao, Jonathan B. McHugh, David C. Miller, Nazanene H. Esfandiari

**Affiliations:** ^1^Division of Metabolism, Endocrinology & Diabetes, Department of Internal Medicine, The University of Michigan, Brehm Tower Room 5107, SPC 5714, 1000 Wall Street, Ann Arbor, MI 48105-1912, USA; ^2^Department of Pathology, The University of Michigan, 1500 E. Medical Center Drive, Room 2G332 UH/Box 0054, Ann Arbor, MI 48109-0054, USA; ^3^Department of Urology, The University of Michigan, Building 16, 1st Floor, Room 108E, 2800 Plymouth Road, Ann Arbor, MI 48109-2800, USA; ^4^Division of Metabolism, Endocrinology & Diabetes, Department of Internal Medicine, The University of Michigan, Domino's Farms, Lobby C, Suite 1300, 24 Frank Lloyd Wright Drive, Ann Arbor, MI 48106-0451, USA

## Abstract

*Objective*. We here report a patient with upper urinary tract urothelial carcinoma with hypercalcemia likely due to elevated 1,25-dihydroxyvitamin D. *Methods*. We present a clinical case and a summary of literature search. *Results*. A 57-year-old man, recently diagnosed with a left renal mass, for which a core biopsy showed renal cell carcinoma, was admitted for hypercalcemia of 11.0 mg/mL He also had five small right lung nodules with a negative bone scan. Both intact parathyroid hormone and parathyroid hormone-related peptide were appropriately low, and 1,25-dihydroxyvitamin D was elevated at 118 pg/dL. The patient's calcium was normalized after hydration, and he underwent radical nephrectomy. On the postoperative day 6, a repeat 1,25-dihydroxyvitamin D was 24 pg/mL with a calcium of 8.1 mg/dL. Pathology showed a 6 cm high-grade urothelial carcinoma with divergent differentiation. We identified a total of 27 previously reported cases with hypercalcemia and upper tract urothelial carcinoma in English. No cases have a documented elevated 1,25-dihydroxyvitamin D level. *Conclusion*. This clinical course suggests that hypercalcemia in this case is from the patient's tumor, which was likely producing 1,25-dihydroxyvitamin D. Considering the therapeutic implications, hypercalcemia in patients with upper urinary tract urothelial carcinoma should be evaluated with 1,25-dihydroxyvitamin D.

## 1. Introduction

Hypercalcemia is one of the most common paraneoplastic syndromes. Although hypercalcemia is found in 13–20% of patients with renal cell carcinoma [1], reports of upper tract urothelial carcinoma complicated by hypercalcemia are sparse. This can be explained partially from the rarity of the disease: accounting for 10% of all renal tumors and 5% of all urothelial malignancies and with roughly 3,000 newly diagnosed cases in the USA in 2007 [2]. Little has been known about the mechanism of hypercalcemia associated with upper tract urothelial carcinoma. Here, we report a case of upper tract urothelial carcinoma, where the cause of hypercalcemia was presumably due to elevated 1,25-dihydroxyvitamin D.

## 2. Case 

A 57-year-old man with a history of hypertension presented with left flank pain, anorexia, and 23 kg of weight loss over two months. He did not have gross hematuria or fever. An abdominal computed tomography (CT) scan revealed a 6 × 4.8 cm mass arising from the medial aspect of the lower pole of the left kidney, with retroperitoneal lymph node enlargement, and tumor nodularity spreading along the anterior pararenal fascia. The left renal vein and inferior vena cava were patent. A core biopsy of the left kidney mass concluded a high-grade, clear cell renal cell carcinoma. Further staging evaluation demonstrated five small right lung nodules by a chest CT scan felt to be metastatic lesions. There was no metabolically active bone lesion on bone scintigraphy.

The patient was scheduled for left radical nephrectomy with retroperitoneal lymph node dissection. His preoperative workup revealed mild hyponatremia, mild hyperkalemia, and hypercalcemia. He complained of nausea without vomiting and constipation for 3 weeks, which he attributed to narcotic use for pain control. He did not have any change in mood. He had no history of kidney stones or fractures. The review of systems was positive for dyspnea on exertion, but otherwise noncontributory. He had no other significant medical history. The family history was unremarkable. He quit smoking 20 years ago and was a social drinker. He was a truck driver.

His medications included atenolol 100 mg daily and lisinopril 5 mg daily, but he was not taking them consistently. His other home medications included acetaminophen/hydrocodone as needed for pain. He had never been on hydrochlorothiazide or any over-the-counter medications or herbal supplements. Prior to admission, he reported fluid intake of approximately 3.5 L per day. 

Physical examination showed a man with a height of 165 cm and weight of 94.1 kg. His vital signs were as follows: blood pressure 135/69 mmHg, pulse 99/minute, respiratory rate 20/minute, and body temperature 36.7°C. He was well developed, well nourished, and without acute distress. He was alert and oriented, and his affect appeared normal. His tongue was moist. His thyroid examination was normal. The chest was clear to auscultation bilaterally. Cardiovascular examination revealed normal S1 and S2 sounds and a regular rate and rhythm without murmur, gallop, or rubs. The abdomen was soft and nondistended, although there was a palpable, fist-sized mass in the left lower quadrant. Bowel sounds were normal. There was no lower extremity edema. A neurological examination was unremarkable. There was no skin rash.

His laboratory data at admission are shown in Table 1. At admission, sodium was 129 mmol/L, potassium 5.2 mmol/L, and calcium 11.0 mg/dL with albumin 3.6 g/dL. Both intact PTH (immunochemiluminometric assay) and PTHrP (immunochemiluminometric assay) were appropriately low for his hypercalcemia. His 1,25-dihydroxyvitamin D level was 118 pg/dL (radioimmunoassay, reference range 18–72), which was inappropriately high for his hypercalcemia. A random cortisol of 34.8 mcg/dL (reference range 3.0–13.0 in the afternoon) ruled out adrenal insufficiency, as a cause of hyponatremia, hyperkalemia, and hypercalcemia. Initially, 0.9% NaCl was given intravenously at 125 mL/hour to correct hypercalcemia. Two days later, serum calcium was normalized and he was discharged. Since his random glucose was elevated a few times, glycated hemoglobin was checked and he was diagnosed with diabetes mellitus for the first time.

**Figure 1:**
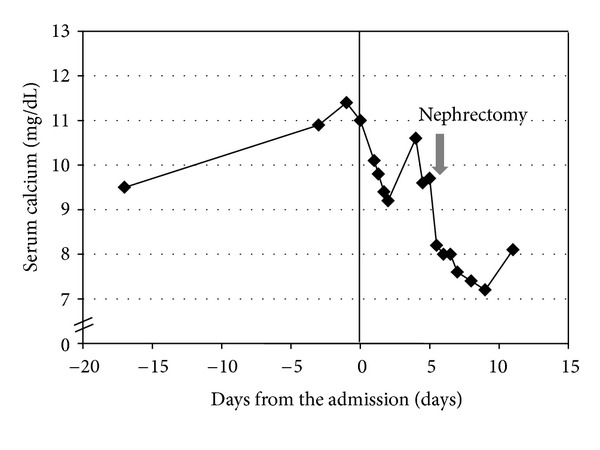
Trends of serum calcium. Note that serum calcium is not corrected for albumin level. Albumin on admission was 3.6 grams/dL, while albumin before the nephrectomy was 2.8 grams/dL.

 The patient was readmitted two days later for his scheduled left open nephrectomy with retroperitoneal lymph node dissection. At that time, calcium was again elevated at 10.6 mg/dL with an albumin of 3.6 grams/dL. The next day, he underwent his scheduled surgery. Intraoperatively, extensive left renal tumor with the involvement of the colonic mesentery, psoas muscle, and retroperitoneal lymphadenopathy was found. Therefore, left radical nephrectomy with left adrenalectomy, retroperitoneal lymph node resection, and en bloc colon resection was performed. Due to the significant blood loss (1,600 mL) during the operation, the patient received 1 unit of packed red blood cell transfusion and stayed in the surgical intensive care unit for a day. Postoperatively, the patient's calcium remained low (Figure 1). On the postoperative day 6, a repeat 1,25-dihydroxyvitamin D showed a level of 24 pg/mL with a calcium of 8.1 mg/dL. He was discharged home on the postoperative day 6. Pathology showed a 6 cm high-grade urothelial carcinoma of the renal pelvis and ureter with divergent differentiation, including 80% clear cell squamous differentiation and 5% sarcomatoid differentiation. The tumor had invaded through the kidney into the perinephric fat. The margin was positive and extensive angiolymphatic invasion was identified as well. Two lymph nodes were examined and both were positive for metastatic carcinoma.

 About three weeks after the discharge, a repeat chest CT scan showed significant increase in size and number of pulmonary nodules. He was referred to a local oncologist for palliative chemotherapy. He died five weeks after discharge.

## 3. Discussion

We report a case of upper tract urothelial carcinoma with hypercalcemia. At the time of hypercalcemia, intact PTH was appropriately suppressed, and PTHrP was not elevated. 1,25-dihydroxyvitamin D, on the other hand, was markedly elevated. The hypercalcemia resolved soon after the removal of the tumor, and the patient's 1,25-dihydroxyvitamin D level decreased to the normal range. This clinical course suggests that the hypercalcemia in this case was caused by the patient's tumor, which was likely producing 1,25-dihydroxyvitamin D.

Because upper urinary tract urothelial carcinoma is relatively uncommon, there is limited information available for the mechanisms of hypercalcemia associated with this type of tumor. 1,25-Dihydroxyvitamin D is a known cause of hypercalcemia in disorders such as lymphoma [28], granulomatous diseases [29, 30], and malignancy [31–33], including renal cell carcinoma [34, 35]. Lee et al. summarized five cases of previously published case reports of humoral hypercalcemia associated with the renal pelvis carcinoma in 1988 [15]. We identified 27 cases previously published in English, in addition to our case (Table 2). To our knowledge, our report presents the first case of hypercalcemia with upper tract urothelial carcinoma with a documented elevated 1,25-dihydroxyvitamin D level. The cases with a suppressed PTH without an elevation of PTHrP [9, 16, 21, 22, 25] could have been from an elevated 1,25-dihydroxyvitamin D if it had been measured. Some other cases [3, 11, 26] might have proven an elevated 1,25-dihydroxyvitamin D, if the full workup was available. 

It is likely that 1,25-dihydroxyvitamin D-associated hypercalcemia is due to the increased conversion of 25-hydroxyvitamin D to 1,25-dihydroxyvitamin D by 1*α*-hydroxylase activity. 1*α*-Hydroxylase was originally thought to be exclusively expressed at the proximal tubules of the kidney [36], although a recent work showed diffuse expression along the nephron [37] and extrarenally [38]. It is our speculation that the tumor in this case overexpressed 1*α*-hydroxylase, resulting in high 1,25-dihydroxyvitamin D levels, but we were unable to stain 1*α*-hydroxylase on the specimen.

The hypercalcemia in this case resolved after the removal of the tumor, which is consistent with most previous case reports. Had our case not been operative, or the hypercalcemia recurred after nephrectomy, glucocorticoid treatment could have been an option.

In addition to hypercalcemia, we make a note that this case had leukocytosis. There are case reports of upper urothelial carcinoma associated with leukocytosis [23, 24], one of which reported the elevation of serum granulocyte colony-stimulating factor (G-CSF) [23]. In our case, leukocytosis might be multifactorial.

In conclusion, we report an uncommon case of hypercalcemia from high 1,25-dihydroxyvitamin D in an upper urinary tract urothelial carcinoma possibly related to the overexpression of 1*α*-hydroxylase, which resolved after nephrectomy. Considering the therapeutic implications, hypercalcemia in upper urinary tract urothelial carcinoma should be evaluated with 1,25-dihydroxyvitamin D.

## Figures and Tables

**Table 1:** Clinical laboratory data.

	Admission	After surgery	Reference range	Unit
Sodium	129	132	136–146	mmol/L
Potassium	5.2	4.7	3.5–5.0	mmol/L
Chloride	92	102	98–108	mmol/L
Bicarbonate	28	25	22–34	mmol/L
BUN	16	10	8–20	mg/dL
Creatinine	0.9	0.7	0.7–1.3	mg/dL
Glucose	152	209	73–110	mg/dL
Calcium	11.0	8.2	8.6–10.3	mg/dL
Phosphorous	4.0	4.4	2.7–4.6	mg/dL
Magnesium	2.3	1.6	1.5–2.4	mg/dL
Albumin	3.6		3.5–4.9	g/dL
Ionized calcium	1.45	1.30	1.12–1.30	mmol/L
WBC	20.7	25.4	4.0–10.0	×10^3^/mm^3^
Neutrophil	87.6	86.2	36.0–75.0	%
Lymphocyte	7.5	8.6	20.0–50.0	%
Monocyte	4.1	3.9	3.0–10.0	%
Eosinophil	0.4	0.8	0.0–4.0	%
Basophil	0.4	0.5	0.0–2.0	%
Hemoglobin	12.6	10.9	13.0–17.3	g/dL
Hematocrit	37.3	32.9	39.0–50.2	%
Platelet	361	499	150–450	×10^3^/mm^3^
Intact PTH	2		10–65	pg/mL
PTHrP	1.1		<2.0	pmol/L
25-Hydroxyvitamin D	51		25–80	ng/mL
1,25-Dihydroxyvitamin D	118	24	18–72	pg/mL
Serum osmolality	276		269–298	mosm/K
Urine osmolality	589		300–1300	mosm/K
Urine sodium	86			mmol/L
HbA1c	8.4		3.8–6.4	%
TSH	1.53		0.30–5.50	mIU/L
Serum cortisol	34.8		3.0–13.0*	mcg/dL

BUN: blood urea nitrogen, WBC: white blood cell.

*Reference range in the afternoon.

**Table 2:** Published cases of hypercalcemia associated with upper tract urothelial carcinoma except for the cases with bone metastasis as a sole potential cause.

Reference	Age, sex	Site	Histology	Calcium (mg/dL)	Phosphorous (mg/dL)	PTH	PTHrP	25-OHD	1,25(OH)_2_D	Lithiasis^(b)^	Concurrent conditions
Bourn et al.,1964 [3]	69 M	P	TR	16.9	3.4	?	?	?	?	++	A shrapnel wound to the kidney
Hodgkinson, 1964 [4]	59 F	P	TR, SQ	16.3	3.7–4.2	?	?	?	?	+	Parathyroid adenoma
Dean et al., 1969 [5]	47 F	P	SQ	13.4	2.4	?	?	?	?		Parathyroid adenoma; a horseshoe kidney
Scully and McNeely, 1974 [6]	68 M	P	TR, SQ	19.7	3.3	> ×2 normal upper limit	?	?	?	+	Bone metastasis; undetectable PTH in the tumor extract
Mandell et al., 1978 [7]	57 M	P, U, B	TR	14.2	3.1	Elevated	?	?	?		
Mandell et al., 1978 [7]	60 F	P, U	TR, SQ	13.5	2.0	Elevated	?	?	?		
Pigadas et al., 1978 [8]	71 F	P, B	TR, SQ	13.3	?	170 pg/mL (ref. 255 ± 92)	?	?	?	++	Hyperplastic parathyroid
Cutshall and Melman, 1979 [9]	64 M	P	TR	14	3.4	Undetectable	?	?	?		Undetectable for PTH in the tumor
Gonzolez et al., 1985 [10]	55 M	P	SQ	13	3	69 mIU per cent^(c)^ (ref. 250–410)	?	?	?	++	Tumor tissue positive for PTH
Harel et al.,1985 [11]	48 M	P	TR	15.3	3.4	?	?	?	?		
Ramsay and Hendry, 1986 [12]	28 M	P	TR	15.2^(a)^	?	Elevated	?	?	?	+	Bone metastasis
Schaefer and Geelhoed, 1986 [13]	58 M	P	SQ	11.3	2.3	840 ng/mL (ref. 430–1816)	?	?	?	+	Parathyroid hyperplasia
Jacqmin et al., 1987 [14]	80 M	P	SQ	13.3	?	Elevated	?	?	?	+	
Lee et al., 1988 [15]	32 F	P	SQ	12.1	2.1	514 pg/mL (ref. 430–1860)	?	?	?	++	
Derbyshire et al., 1989 [16]	45 M	P	TR	12.0^(a)^	?	0.53 ng/mL (ref. <1.0)	?	?	?	+	Analgesic nephropathy
Derbyshire et al., 1989 [16]	45 F	P	TR	12.8^(a)^	?	<0.2 ng/mL (ref. 0.2–0.6)	?	?	?	+	Analgesic nephropathy
Castillo et al., 1991 [17]	67 F	P	TR, SQ	14.6^(a)^	?	Normal	?	?	?		
Sandhu et al., 1991 [18]	60 M	P	SQ	11.2^(a)^	?	2.8 pmol/L (ref. 0.8–8.5)	?	?	?		History of papillary tumor of the bladder
Lee et al., 1994 [19]	53 M	P	TR	13.6	2.6	Normal	?	6.0 ng/mL	6.0 pg/mL	++	Coexisting ipsilateral renal cell carcinoma
Matsuoka et al., 1994 [20]	78 M	U	TR	13.9^(a)^	3.9	<3 pg/mL (ref. 15–50)	?	?	?		Elevated urinary PTHrP; positive for PTHrP staining on metastatic lesion
O'Sullivan et al., 1994 [21]	78 M	P	SQ	13.8	Low normal	Undetectable	?	?	?	++	History of tuberculosis
Cadeddu and Jarrett, 1998 [22]	67 F	P	SQ	11.7	?	3 pg/mL (ref. 10–65)	?	7 ng/mL (ref. 10–55)	?	++	
Kamai et al., 1998 [23]	53 M	P	SQ	19.0^(a)^	?	?	12.0** **pmol/L (ref. <1.1)	?	?		Positive for PTHrP staining on the tumor
Er et al., 2001 [24]	58 M	P	SQ	14.4	5.3	28 pg/mL (ref. 9–55)	?	?	?	++	
Grubb et al., 2004 [25]	44 F	P	TR	13.6	?	<5 pg/mL (ref. 14–72)	?	?	?		Polycystic kidney disease
Li et al., 2007 [26]	49 M	P, U	?	Elevated	?	?	?	?	?		Bone-marrow metastasis
McMahan and Linneman, 2009 [27]	71 M	U	TR	14.4	?	Normal	49.5 pmol/L (ref. 0–4.7)	?	?		
Present case	57 M	P, U	TR, SQ, SC	11.0	4.0	2 pg/mL (ref. 10–65)	1.1 pmol/L (ref. <2.0)	51 ng/mL (ref. 25–80)	118 pg/mL (ref. 18–72)		

^(a)^Corrected calcium; ^(b)^++ staghorn calculus, + other lithiasis; ^(c)^C-terminal PTH. Site: P (renal pelvis), U (ureter), and B (bladder). Histology: TR (transitional), SQ (squamous, including squamous metaplasia), and SC (sarcomatoid). 25-OHD: 25-hydroxyvitamin D, 1,25(OH)_2_D: 1,25-dihydroxyvitamin D, ?: not mentioned; and ref.: reference range.
